# Biofilm spatial structure and superinfection immunity modulate inter-phage competition

**DOI:** 10.1371/journal.pbio.3003737

**Published:** 2026-03-31

**Authors:** James B. Winans, Carey D. Nadell

**Affiliations:** 1 Department of Biological Sciences, Dartmouth College, Hanover, New Hampshire, United States of America; 2 Department of Microbiology and Immunology, Geisel School of Medicine at Dartmouth, Hanover, New Hampshire, United States of America; Monash University, AUSTRALIA

## Abstract

Obligately lytic (virulent) phages always lyse host cells to release progeny viruses, while temperate phages can either lyse their hosts or integrate into host genomes as prophages, forming lysogens. There is a rich history of work studying the relative advantages and disadvantages of these two phage life history strategies, but little of this work has addressed the spatial constraints common to biofilm environments. We developed a live imaging system to track lytic infections, lysogenic infections, and uninfected cells at single-cell resolution within three-dimensional *Escherichia coli* biofilms. We find that biofilm structure substantially impacts the ecological success of different phage infection strategies. Temperate phages have the unique capacity to release phages from lysogens that have undergone lytic induction from within the interior of mature biofilms. When this occurs in biofilm contexts that do not limit phage diffusion, lytic infections expand rapidly, but lysogenic infections are favored as phage mobility declines in densely packed biofilm architectures. In matrix-replete biofilms that do limit phage mobility, lytic phage infection is more limited, favoring lysogenic growth. Direct competition assays between lysogenized host bacteria and obligately lytic phages—with or without the ability to superinfect lysogens—revealed that spatial structure and superinfection potential together greatly impact phage competition outcomes during co-invasion into pre-existing, phage-susceptible biofilm populations. Highly packed, phage diffusion-impeding biofilms disproportionately favored temperate phages in the lysogenic cycle over obligate lytic phages, highlighting how biofilm architecture can constrain lytic phage infection and promote vertical phage genome transmission strategies.

## Abstract 2

Bacteriophages vary widely in life history strategy, but little is known about how these strategies influence host invasion and inter-phage competition in biofilms. This study explores the abilities of temperate lysogens and virulent phages within biofilms, showing that spatial structure shapes invasion success, favoring temperate phages when diffusion is limited.

## Introduction

Temperate and obligately lytic (virulent) phages follow distinct life history strategies [1,2]. Virulent phages inject their genome into a host cell, commandeer their hosts’ replication machinery, and rapidly lyse the cell to release progeny virions [3,4]. Temperate phages can choose to follow the same lytic pathway, or alternatively they can integrate their genome into that of the host as a prophage, converting the cell into a lysogen [5–7]. In both cases, the phage co-opts host resources to propagate, but while lytic infections result in imminent cell death, prophages are often less intrusive to their hosts’ replication, transmitting the phage genome through vertical inheritance until reentry into the lytic cycle [8–10]. Classic theory and more recent work have explored the fitness tradeoffs incurred by these distinct phage strategies, examining how host availability, multiplicity of infection, and environmental conditions influence the success of lysis versus lysogeny in competition [11–18]. However, experimental work that addresses competition for host cells between lytic and temperate phages has focused primarily on bulk liquid culture, rather than on surface-attached groups (biofilms) that bacteria often occupy [18–22]. Recent studies have illustrated that when host bacteria secrete extracellular matrix and grow into biofilm cell clusters, they can collectively slow or halt phage diffusion amongst them [23–31].

Previously, we explored in detail how temperate phage λ*cI*_857_ interacts with biofilm-dwelling host populations of *E. coli*, finding that if host biofilm matrix production and corresponding architecture are established, then λ*cI*_857_ virions can infect hosts along the biofilm exterior but are unable to access hosts on the biofilm interior [24]. As a consequence, λ*cI*_857_ lysogens arise along the biofilm periphery, where they are predisposed to disperse back into the surrounding environment, which itself can lead to over-representation of λ*cI*_857_ lysogens in new biofilms generated in downstream locations [24]. Here we build on this work by interrogating whether phages spontaneously released by lysogens from within the biofilm interior can mobilize to infect new hosts effectively and thereby alter resident biofilm structure over time. Furthermore, we aimed to assess how λ*cI*_857_ lysogens and virulent λ derivatives might compete with one another in the process of invading a biofilm of susceptible hosts with variable matrix secretion. We approached this question with the simple hypothesis that host genome-embedded prophages and phage virions would have different capabilities to spread when released within biofilms, and also different abilities to invade pre-existing biofilm populations. If supported, this would indicate that the spatial constraints and host physiology specific to biofilm environments are important to consider for the evolutionary dynamics of temperate versus virulent bacteriophage strategies.

We developed an experimental system allowing for live imaging of λ phage particles, lytically infected cells, lysogenized cells, and uninfected host cells, all differentiated by distinct fluorescent reporters within biofilm communities grown under continuous flow [24]. Using this system, we explore two general scenarios under which temperate phages λ might interact differently with host cells in the biofilm context relative to virulent phages of otherwise similar infection characteristics (λvir and λΔ*cI*). First, temperate phages have the distinguishing ability to release phages from within the interior of biofilms in which lysogens have become embedded. We test here how effectively phage virions can spread from biofilm-embedded lysogens undergoing lytic activation, and how their propagation depends on host biofilm architecture. Second, lysogenized host cells and phage virions—due to differences in size, surface properties, and presence/absence of active motility—may differ in their ability to invade biofilms from their exterior, which in turn may also depend on biofilm architecture. To directly compete the two phage lifestyles, we co-invade lysogenized cells and lytically locked phages that either could or could not successfully superinfect lysogens (λvir and λΔ*cI*, respectively) into biofilm communities. By introducing isogenic phages with these different infection properties into biofilms of varying architecture and tracking their spatial propagation over time, we explore how phage life history strategy, superinfection potential, and spatial structure collectively shape the competitive ability of distinct phage life histories in the biofilm context.

## Results

### Biofilm structure modulates spread of phages released from lysogens embedded within biofilms

A potential advantage distinct to temperate phages in the biofilm context is the ability to disseminate from lysogens present early during biofilm growth and embedded in the interior of the host population [24]. To investigate under what conditions this potential advantage occurs, we established lysogen-embedded *E. coli* biofilms in curli-producing (WT AR3110, denoted curli^+^) or curli non-producing (Δ*csgBA*, denoted curli^−^) *E. coli* strain backgrounds. In prior work, while testing for pleiotropic effects of the Δ*csgBA* deletion, we did not detect any physiological differences between the parental AR3110 strain and its Δ*csgBA* derivative with respect to λ phage plaquing ability, adsorption rate, or phage titer population dynamics in well-mixed liquid culture [24]. In biofilm environments, secreted curli polymer matrix, and the multicellular architecture associated with it, greatly reduces phages’ ability to diffuse into biofilms from their exterior [23–25]; however, it is less clear how phages might disseminate if released from lysogens that co-colonized a surface with uninfected cells, leading to mixed-strain populations with lysogens present on the interior.

**Fig 1:**
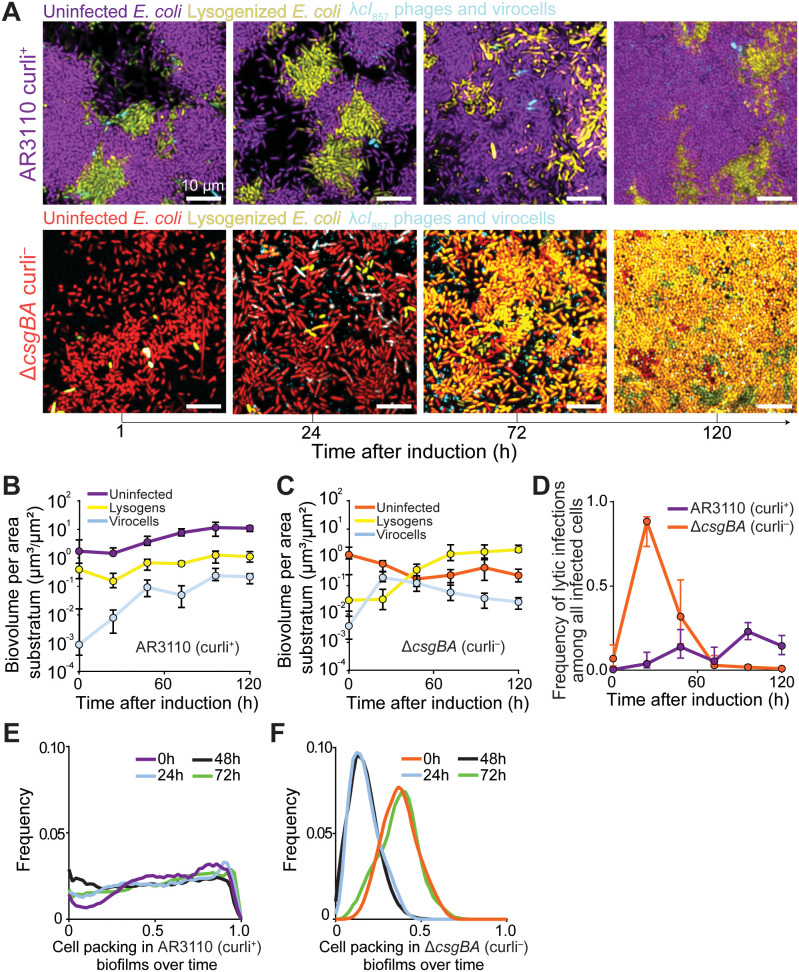
λ*cI*_857_ lytic infections and host lysogenization following induction of lysogens embedded within *Escherichia coli* biofilms with either WT curli production (curli^+^) or no curli production (curli^−^). Host biofilms were inoculated with λ*cI*_857_ lysogens and uninfected host cells at a 1:10 ratio and cultured for 72 h prior to λ*cI*_857_ phage induction. **(A)** Time series of biofilms inoculated with lysogens and uninfected *E. coli* hosts that either produce (top row) or do not produce (bottom row) curli extracellular matrix. The first image in each row was taken just after heat was applied to induce some biofilm-embedded lysogens to switch to the lytic infection cycle. **(B, C)** Population dynamics of active lytic infections, lysogens, and uninfected host cells in curli^+^ and curli^−^ biofilms (*n* = 7–24). **(D)** Population dynamics of infected cells that are currently virocells (in the lytic pathway) in curli^+^ and curli^−^ biofilms (*n* = 7–24). **(E, F)** Time-resolved cell packing frequency distributions within curli^+^ and curli^−^ biofilms. For panels B–D, center points denote medians, and bars denote interquartile ranges. All figure panels are new analyses derived from data published in Winans and colleagues (2025) [24]. The numerical data within this Figure can be found in S1 Data.

We first grew *E. coli* biofilms comprised of uninfected *E. coli* and *E. coli* lysogenized with λ*cI*_857_ at an inoculation ratio of 10:1. In one set of experiments, both uninfected and infected cells were curli^+^; in a second set of experiments, both strains were curli^−^. Our primary model λ phage harbors the temperature-sensitive *cI*_857_ allele, such that lysogens are relatively stable and spontaneously induce to the lytic cycle at a rate of 10^−5^ in our room temperature biofilm culture conditions (S1 Fig). Because the total bacterial count in our culture chambers was on the order of 10^6^, spontaneous lytic induction by λ*cI*_857_ lysogens and subsequent phage spread did not occur frequently enough to be detected without experimental intervention [24]. After 72 h of biofilm growth, we therefore induced some but not all lysogenized cells to enter the lytic cycle via a short heat treatment, after which cultures were returned to room temperature (see Methods). This resulted in 1% and 7% lytic induction in the curli^+^ and curli^−^ biofilm contexts, respectively (S2 Fig). We then tracked these systems with daily imaging for 120 h (Fig 1A–1C). To assess population dynamics and compare the relative abilities of phages versus lysogens to replicate—and in keeping with recent theory [32,33]—we quantify lysogens and virocells (i.e., host cells undergoing productive lytic phage infection) as the primary fitness metrics. The absolute number of lysogens in these experiments initially declined following lytic induction and then increased in abundance thereafter (Fig 1B).

In biofilms of the AR3110 (curli^+^) background, the susceptible host population was mostly protected from phage exposure and maintained net positive growth (Fig 1B). Lytic infections were visible at low frequency; at maximum, they represented less than 5% of the total cell population, and among all infected cells (virocells and lysogens), lytic infections peaked at ~15% by 96 h and then later declined again (Fig 1D). The results indicate that temperate phage virions can indeed spread when released within the interior of curli-producing *E. coli* biofilms, albeit to a limited extent. This observation is consistent with previous reports, which showed that growing bacterial populations can support a lytic phage population, dependent on spatial constraints [23,25,34]. In curli^−^ biofilms, on the other hand, virocell frequencies rose within 24 h after λ*cI*_857_ induction to ~88% of the total infected cell population (Fig 1C and 1D). Thereafter, as lysogen counts increased sharply, virocell biovolume consistently declined due to the depletion of available uninfected, phage-sensitive hosts. Lysogen counts surpassed uninfected host cells by 48 h and ultimately comprised the majority of the bacterial population (Fig 1A and 1C).

As noted above, our primary model phage λ carries the *cI*_857_ allele, which removes cI Repressor’s wild type sensitivity to host RecA and instead confers temperature sensitivity to cI; this allowed us to control the timing of lytic induction via heat treatment. To ensure that the results above were representative of spontaneous lytic induction by true wild-type λ phages with cI RecA sensitivity, we reconstructed the wild-type RecA-sensitive allele in the parental λ strain, such that it still contained the fluorescent protein constructs required for visualizing host lysogenization and phage virions (this strain is denoted λ*cI*_WT_). We then repeated the experiments depicted in Fig 1B and 1C and found the same qualitative population dynamics for phages, lysogens, and uninfected host *E. coli* when embedded in both AR3110 (curli^+^) and Δ*csgBA* (curli^−^) host populations. The main difference between the results for λ*cI*_WT_ and those shown in Fig 1B and 1C was the timing of phage propagation and lysogen spread in the case of the Δ*csgBA* host biofilm context (S3 Fig).

The two distinct patterns of phage spread in AR3110 curli^+^ versus Δ*csgBA* curli^−^ populations imposed correspondingly distinct feedback on the architecture of the biofilms in which they were occurring. Because phage-mediated killing and subsequent lysogen regrowth are expected to directly restructure local cellular crowding, we measured cell packing frequency distributions at 1-day intervals following the heat induction step of these experiments. In the AR3110 curli^+^ *E. coli* background, the frequency distribution of cell packing changed negligibly over the course of induction and subsequent phage spread, reflecting the limitation on phage diffusion and new lytic infections imposed by curli-replete biofilm architecture (Fig 1E). In the curli^−^ background, by contrast, the whole population was down-shifted for cell packing density distribution due to the large fraction of cells infected and killed by phages released from induced lysogens. After most of the curli^−^ population was lysogenized, the biofilms then recovered to their original cell packing distributions (Fig 1F).

We were curious as to whether the results above could be due to reduced expression of the λ phage receptor (LamB maltoporin) by *E. coli* in the AR3110 curli^+^ population, which reaches larger absolute size and cell packing than the Δ*csgBA* curli^−^ background. To control for this possibility, we produced an sfGFP transcriptional reporter for the *mal* operon (see Methods). When maltose is supplied as the sole carbon source, as was the case for all main text experiments, the *mal* operon reporter was active in the large majority of the biofilm population, regardless of spatial location. Variation in phage receptor expression therefore cannot explain the greatly reduced rate of lytic and lysogenic infection in curli^+^ experiments (S4 Fig).

Altogether, these results demonstrate that temperate phages can indeed propagate after induction of biofilm-embedded lysogens, but the fraction of host cells accessible to released phage virions is constrained by the presence of extracellular matrix and the packing architecture that matrix confers to host cells. In curli^+^ biofilms, impeded diffusion of phage particles limits widespread infection, and sensitive cells remain in the majority among host *E. coli*. In curli^−^ biofilms, where phages can diffuse more freely, phages released from induced lysogens cause a rapid increase in new infections throughout the population. While phages in the lytic cycle temporarily outnumber phages in the lysogenic cycle, a decrease in sensitive cells and lysogenic cell accumulation leads to a drop in virocell frequency over time and longer-term dominance of the lysogen sub-population.

### Biofilm structure and superinfection immunity shape temperate and virulent phage propagation during invasion into pre-established biofilms

We next investigated how temperate and virulent λ phages compete during co-invasion into pre-formed *E. coli* biofilms. In particular, we compared the biofilm invasion abilities of temperate phage λ*cI*_857_ lysogens and λ phages genetically restricted to the lytic cycle, including λvir (able to infect lysogens) and λΔ*cI* (unable to infect lysogens). Both phages can adsorb to host bacteria lysogenized by λ, but λΔ*cI* is suppressed following genome injection [35,36]. The rationale for co-invading λ*cI*_857_ lysogens with virulent λ phages was to explore the potential advantages and disadvantages of being embedded within a bacterial host—which in principle allows temperate phages to take advantage of native host physiology that they do not themselves encode—when invading a pre-existing biofilm population. Depending on resident biofilm architecture, other bacteria may or may not be able to invade and take hold within the resident population, as has been explored previously [26,37,38]. Phage particles also display variable biofilm invasion and infection potential as a function of phage virions’ biophysical properties and host biofilm architecture [24,25,28,39]. Co-invasion of resident biofilms by both lysogenized cells and virulent phages adds complexity to the invasibility question, as both the invading phages and lysogens can potentially change the composition and architecture of the resident biofilm environment. Whether or not virulent phages can superinfect co-invading lysogens is another important parameter to explore for this question, and to accommodate it we performed all of the experiments below (in separate treatments) with either superinfecting virulent phages (λvir) or non-superinfecting virulent phages (λΔ*cI*).

In separate experiments, we performed phage and lysogen co-invasions into *E. coli* biofilms formed by strains with no curli production (Δ*csgBA*, denoted curli^−^), wild type curli production (AR3110 parental strain, denoted curli^+^), or curli overexpression (csgD*, denoted curli^++^); the three host strains display no significant difference in their efficiency of plating for any of the λ phages used here (S5 Fig). We grew biofilms of these strains for 72 h prior to introducing equal titers of virulent phages and λ*cI*_857_ lysogens (10^4^ [PFU/CFU]µL^−1^ for 2 h), after which biofilms were imaged daily for 120 h to quantify phage and lysogen proliferation, as well as uninfected host population dynamics (S6 Fig). The 72 h cultivation period prior to phage/lysogen invasion was chosen because it allows for biofilm matrix production to a steady state for these culture conditions. Additionally, the biofilms produced by *E. coli* in this time frame do not become large enough for metabolic stratification to occur via growth substrate depletion on the biofilm interior [40–42]. This mitigates the concern that bacteria on the biofilm interior might be less susceptible to infection due to stalled metabolic activity.

The introduced lysogens were in the AR3110 (curli^+^) background in all cases for consistency across treatments, and we confirmed in earlier work that introducing a minority population of curli^+^ cells does not substantially alter resident biofilm architecture on the time scale of these experiments [27,39]. To control for the possibility of phage-lysogen interaction within the syringes during the 2 h invasion period, we performed separate experiments in which the invasion inoculum mixtures (λ*cI*_857_ lysogens with λvir phages; or λ*cI*_857_ lysogens with λΔ*cI* phages) were sampled every 15 min for 2 h; these controls revealed no substantial changes in lysogen or phage titer over the 2 h time period (S7 Fig).

The central motivating questions for these experiments were (1) whether it is more favorable under direct competition to invade a pre-existing biofilm as a virulent phage particle or as a prophage inside a host, and (2) whether the answers to the first question change as a function of host biofilm architecture and/or superinfection exclusion status of the lysogens.

**Fig 2:**
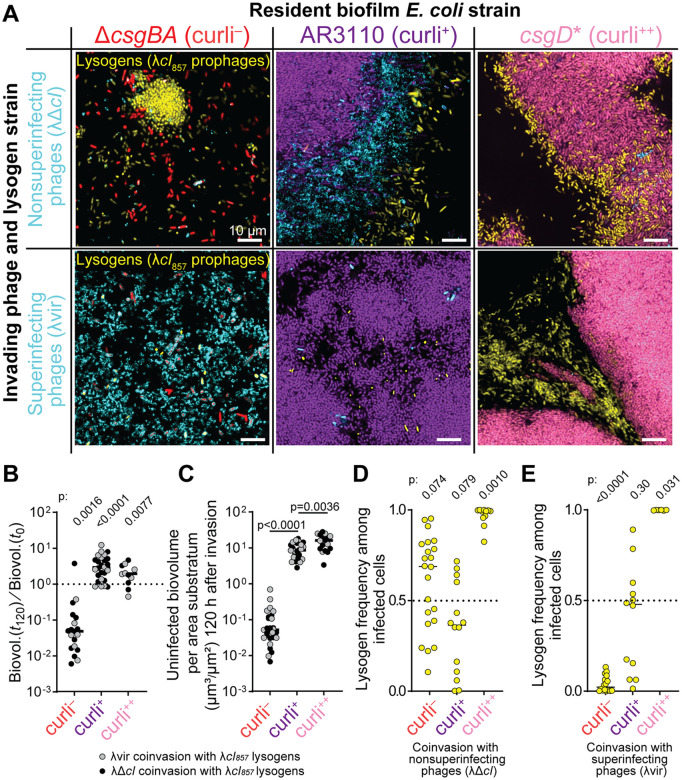
Invasion of resident, λ-sensitive biofilms with λ*cI*_857_ lysogens and virulent derivative phages that either could not superinfect lysogenized hosts (λΔ*cI*) or could superinfect lysogenized hosts (λvir). **(A)** Representative images of biofilms at 120 h after invasion of non-superinfecting phages (top row) or superinfecting phages (bottom row) and lysogens into curli^−^ (left column), curli^+^ (middle column), or curli^++^ (right column) biofilms. **(B)** Proportional change in uninfected *E. coli* population sizes for curli^−^, curli^+^, and curli^++^ biofilms at the end of the 120 h experimental monitoring period (*n* = 12–14). *P*-values denote the outcome of Wilcoxon tests against a null prediction of 1.0, which would indicate no net change in uninfected host cells over the course of the experiments. **(C)** Absolute quantification of uninfected *E. coli* at 120 h for each of the three matrix-producing strain variants. *P*-values denote the outcome of Mann-Whitney U tests for pairwise comparisons against curli^+^, which represents wild type curli regulation (*n* = 12–14). **(D)** Frequency of lysogens among phage-infected cells when co-invaded into curli^−^, curli^+^, or curli^++^ biofilms with virulent λΔ*cI* phages that cannot superinfect lysogens. *P*-values denote the outcomes of Wilcoxon tests against a median value of 0.5, which would indicate neutral competition between λ*cI*_857_ lysogens and λΔ*cI* virocell infections (*n* = 11–22). **(E)** Frequency of lysogens among phage-infected cells when co-invaded into curli^−^, curli^+^, or curli^++^ biofilms with virulent λvir phages that can superinfect lysogens. *P*-values denote the outcomes of Wilcoxon tests against a median value of 0.5 (*n* = 6–16). The numerical data within this Figure can be found in S1 Data.

We observed phage invasion into all 3 *E. coli* biofilm variants (curli^−^, curli^+^, curli^++^), but to greatly varying degrees and with different relative success of lysogens and phage virions depending on both biofilm architecture and lysogen superinfection exclusion (Fig 2A). Consistent with prior reports [23,25], the addition of lytic phages to the curli^−^ strain caused the biomass of the resident biofilm to decrease, while curli^+^ and curli^++^ biofilms maintained net positive growth after phage addition (Fig 2B and 2C). To assess competition between λ*cI*_857_ prophages and virulent phages, we focused on the sub-population of host *E. coli* that were lysogenized or undergoing lytic cycle infection. This sub-set of bacterial host cells represents the resource pool that was available to phages over the course of the experiments: we monitored the virocell infected fraction, indicating lytic phage reproduction, and the lysogenized fraction, indicating temperate prophage reproduction. Note again that in these experiments, the spontaneous lytic induction rate of temperate λ*cI*_857_ is below detection, so we are effectively asking whether phage genomes embedded within host cells compete better during biofilm invasion than the virion particles of virulent λ derivatives, which must pass from one lytic host infection to another in order to propagate. We used this approach to determine whether virulent or temperate phage strains best competed for host access, dependent on the cell packing architecture of host biofilms as well as the superinfection exclusion status of lysogenized hosts.

When λ*cI*_857_ lysogens and λΔ*cI* phages were invaded together into curli^−^ or curli^+^ biofilms, outcomes were variable, but on average the λ*cI*_857_ lysogens and λΔ*cI* virocells were both present among phage-infected hosts (Fig 2A, top row and 2D). By contrast, λ*cI*_857_ lysogens represented nearly 100% of actively replicating phage genomes in the majority of cases when invading matrix-overexpressing csgD* (curli^++^) populations; λΔ*cI* virocells were sometimes present but always rare, indicating minimal host accessibility (Fig 2D). When λ*cI*_857_ lysogens and λvir phages were invaded together, the phage-host population dynamics changed, especially when host biofilms were Δ*csgBA* (curli^−^), allowing for free phage diffusion (Fig 2A, bottom row and 2E). When the dual invasion was performed into curli^−^ populations, we observed very few lysogens and a large majority of lytic λvir infections by the end of the experiment (Fig 2E). This result follows from the simple logic that when host biofilm structure poses no barrier to phage diffusion, and virulent phages can super-infect lysogens, the majority of *E. coli* (lysogenic or not) will be killed by lytic infections. There was no systematic difference in the relative replication success of λ*cI*_857_ lysogens and λvir infections when the two were co-invaded into AR3110 (curli^+^) *E. coli* host biofilms, similar to what we observed for co-invasion of λ*cI*_857_ lysogens and λΔ*cI* phages (Fig 2E). Likewise, when invading curli^++^ matrix-overexpressing host biofilms, λ*cI*_857_ lysogens constituted nearly 100% of the phage-infected host cells in all runs of the experiment.

## Discussion

We aimed in this study to assess how temperate lysogens and virulent phages might differ in their abilities to interact with host bacteria in biofilm environments, particularly when introduced together to the same biofilm population of susceptible hosts. Temperate phages have the capacity to burst from a lysogenized host that is already inside the biofilm interior; we showed here that this capacity translates to new host infections and lysogenization events, contingent on whether host biofilm structure impedes phage diffusion. Further, direct competition between temperate lysogens and virulent phages during co-invasion into biofilms has distinct outcomes that also depend on the extent of phage diffusion limitation, as well as lysogens’ superinfection exclusion status. In matrix-deficient Δ*csgBA* (curli^−^) biofilms—which do not substantially impede phage diffusion—temperate lysogens with superinfection exclusion and virulent phages were able to colonize and propagate in comparable quantities. On the other hand, lysogens were always outcompeted by superinfection-capable virulent phages in curli^−^ biofilm settings. On the time scales assessed here, interestingly, AR3110 (curli^+^) biofilms generally supported coexistence of lysogens and virulent phages, both superinfecting and non-superinfecting. Finally, matrix over-expressing csgB* (curli^++^) biofilms strongly favored host-embedded temperate lysogens over virulent phages, regardless of superinfection ability.

A central motivation for this work was the notion that temperate prophages that are replicated along with host cell division also passively benefit from the other host features—for example, dedicated structures for motility and surface attachment—that might be particularly helpful for surviving in biofilm environments. However, another key consideration is that lysogens often require more time for nutrient uptake to grow and divide, whereas virulent phages may replicate more rapidly while depending on access to susceptible hosts for successive rounds of lytic infections. Our results support the intuition that when phage mobility is high and lysogens are not immune to superinfection, virulent phage strategies are particularly successful during biofilm invasion. By contrast, when hosts are tightly packed together within biofilms and phage mobility is low, temperate lysogens are relatively more effective at invading uninfected host populations, regardless of superinfection exclusion. Biofilm populations with intermediate or locally variable phage mobility allowed for invasion of both virulent phages and temperate lysogens. These outcomes are all consistent with the original idea that biofilm structure can alter the relative fitness of virulent versus temperate phages, but future study will be required for establishing what role biofilm-type environments might play more generally in the evolutionary dynamics of temperate versus lytic phage life history strategies. Allowing for repeated rounds of biofilm growth, phage exposure, host and phage dispersal, exit from and re-entry into lysogeny by temperate phages, and variation in the amount of time phages and hosts spend in well-mixed planktonic versus biofilm conditions are all likely to be important for understanding how and why biofilm growth might be important to consider for phage life history evolution.

A key distinction between temperate and virulent phages lies in temperate phages’ ability to actively regulate whether they propagate via vertical transmission through lysogenized host replication or horizontal transmission through lytic infection events, choosing between lysis and lysogeny in response to environmental cues [43–45]. However, in our study, the direct competition experiments involved phages that were locked into either the lytic or lysogenic pathway, a deliberate choice here to clarify the outcome of the experiments. Future research can build on these findings with experiments on longer time scales that allow for temperate phages to switch back and forth between lytic and lysogenic propagation—all while in direct competition with virulent phages that only replicate via lytic infection. Our work here offers a start to this research direction by demonstrating that spatial structure and superinfection immunity can shift the outcome of competition between lysogens and obligately lytic phages.

## Methods

### Strain construction

The *E. coli* strains used in this study were are derivatives of AR3110, which was generated from the K-12 parental strain W3110 by restoration of its biofilm matrix regulation relative to the domesticated ancestor [46]. λvir was generated by introducing three previously identified point mutations that lock λ into the lytic pathway upon host infection and render it insensitive to silencing by λ prophages (i.e., bypassing superinfection immunity) [36]. λΔ*cI* was made via deletion of the *cI* locus encoding Repressor, locking this phage strain into the lytic pathway as well, but without bypassing prophage superinfection immunity. λ*cI*_WT_ was constructed by heat-inducing a strain lysogenized with λ*cI*_857_ to activate the Red genes prior to transforming in a DNA fragment that encodes *cI*_WT_ [47]. Transformed cells were rescued in 5 mL of SOC media overnight at 30°C. Then 500 µL of the rescued cells were back-diluted into 30 mL of LB media and incubated at 30°C for 3 h prior to treatment with Mitomycin C at a concentration of 2.5 µgmL for 3 h. Mitomycin C treatment selected for prophages that are sensitive to the host SOS response and temperature-insensitive. This culture was then centrifuged to pellet lysogens, and the supernatant was filter sterilized to remove any remaining bacterial cells. Phages in suspension were plated for plaque formation. Resultant plaques were then screened for *cI*_WT_ by sequencing. The malK reporter plasmid was produced by placing the malK promoter region upstream of sfGFP, and transformed into designated strain backgrounds. All strains constructed for this study are listed in Table 1 and will be provided upon request.

**Table 1:** Bacterial strains, phage strains, and software packages used in the study.

Strain	Relevant markers/Genotype	Source
Bacteria		
CNE365	AR3110, with point mutation inside csgD promoter according to ref [23], Ptac _mRuby2 and KanR inserted at attB site (Ptac without operator).	[23]
CNE863	ptac-mKatex2::LacI-LacZ. 6xHIS csgA fusion, with AmpR gpD plasmid	[24]
CNE866	mTurquoise/mKo λ*cI*_857_ strain in AR3110 with 6xHIS csgA for curli tagging + GpD Amp Plasmid	[24]
CNE869	ptac-mKatex2::LacI-LacZ csgBA deletion AmpR gpD Plasmid	[24]
CNE913	AR3110 with ΔcsgB::scar lysogenized with mKO/mTurq λ *cI*_857_	[24]
CNE949	AR3110 (curli^+^) holding pSC101 plasmid with *malK* promoter sequence controlling GFP reporter	This study
CNE986	6xHIS-csgA (curli^+^) lysogenized mKO-Lambda with *cI*_WT_, temperature insensitive, SOS sensitive	This study
CNE987	Δ*csgBA* (curli^−^) lysogenized mKO-Lambda with *cI*_WT_, temperature insensitive, SOS sensitive	This study
CNE998	csgD* (curli^++^) holding pSC101 plasmid with *malK* promoter sequence controlling GFP reporter	This study
CNE999	Δ*csgBA* (curli^−^) holding pSC101 plasmid with *malK* promoter sequence controlling GFP reporter	This study
Phages		
λLZ1367	λD-mTurquoise2 λ*cI*_*857*_-mKO2 bor::CmR (λ*cI*_857_)	[7]
CNX20	λD-mTurquoise2, truncated cI1-17::223-239, bor::CmR (λΔ*cI*)	[24]
CNX21	*λD-mTurquoise2, virulent by introducing mutations v1 v2 and v3, bor::CmR* (λvir)	This study
CNX24	*λD-mTurquoise2, cI*_WT_*, bor::CmR, SOS responsive heat unresponsive* (λ*cI*_WT_)	This study
**Software and Algorithms**	**Source**	**Version**
Zen Blue	Zeiss	v3.4.91.00000
MATLAB	MathWorks	vR2021a
BiofilmQ	[48]	v0.2.2

### Microfluidic device fabrication

Microfluidic flow chambers were fabricated by casting poly-dimethylsiloxane (PDMS, Dow SYLGARD 184) onto prepared device molds (schematic provided in S8 Fig). PDMS molds were trimmed, inlet/outlet ports punched, and then bonded to plasma-cleaned #1.5 glass coverslips (Azer Scientific, cat. #1152260). Internal chamber dimensions were 5,000 μm × 500 μm × 70 μm (*L* × *W* × *H*). Media flow connections were established using inlet tubing (Cole Parmer PTFE #30) connected via 27-gauge needles (BD Precision) to 1 mL syringes driven by Harvard Apparatus syringe pumps. Outlet tubing was taped to a waste petri dish to collect chamber effluent.

### Biofilm culture conditions

Non-lysogenic *E. coli* AR3110 strains were grown overnight at 37°C with shaking in LB media; strains lysogenized with λ*cI*_857_ were cultured overnight at 30°C due to temperature-sensitive repressor proteins. Overnight cultures were normalized to OD_600_ = 1.0 before chamber inoculation. After an initial 45-min static incubation to facilitate bacterial attachment, chambers were perfused continuously with M9 minimal medium supplemented with 0.5% maltose at 0.1 μL/min. To test for *mal* operon expression, strains holding the *malK-sfGFP* plasmid were grown in either M9 with 0.5% glucose or M9 with 0.5% maltose, and sfGFP fluorescence was measured as a function of cell packing in both media conditions. All biofilm experiments were performed at room temperature.

### Lytic induction and spontaneous induction assays

To measure phage burst size, lysogenic *E. coli* was plated for CFU counts before and after lytic induction, and the total amount of phages produced by one round of induction was measured by PFU plating. To measure spontaneous induction of lysogenic strains within biofilms, monocultures of λ*cI*_WT_ AR3110 and λ*cI*_857_ AR3110 lysogens were incubated for 72 h and then measured for frequency of lysogens producing the mTurquoise phage capsid label. This assay was performed in monocultures, so that phages produced by spontaneous induction did not lead to further phage infection and replication. For temperate phage induction within biofilms, microfluidic devices containing susceptible host *E. coli* and *E. coli* lysogenized with λ*cI*_857_ (10:1 ratio) were incubated for 72 h prior to lytic induction. The Repressor protein of λ*cI*_857_ variant used in this study is temperature-sensitive. To induce some of the lysogens into the lytic cycle, we incubated microfluidic devices at 42°C for 40 min, which results in ~1%–10% of the lysogen population converting to lytic cycle replication. Following heat treatment, biofilms were monitored for 120 h to quantify the population dynamics of uninfected susceptible cells, lysogenized cells, and virocells undergoing lytic infection. For comparison with lysogens carrying the λ*cI*_WT_ prophage, we repeated this co-culture experiment with susceptible host *E. coli* and *E. coli* lysogenized with λ*cI*_WT_ and monitored the cocultures daily for 168 h without any heat treatment or other experimental manipulation.

### Phage propagation and efficiency of plating

λ*cI*_857_ phages were generated from lysogenized *E. coli* via heat shock at 42°C, followed by incubation at 37°C until host cell lysis. λ*cI*_WT_ phages were prepared by treating lysogenized *E. coli* with 2.5 µgmL of mitomycin C followed by incubation until host cell lysis. Obligately lytic phages (λΔ*cI* and λvir) were generated by inoculating 250 µL of *E. coli* cultured overnight in λ broth with a −80°C glycerol strain stock, incubating for 30 min prior to adding 4.75 mL of fresh λ broth to allow for bacterial growth and phage infection and replication to occur. Phage titers were determined by standard plaque assays and back-diluted to 10^4^ PFU/μL in M9 maltose media for experiments. To test whether the different bacterial strains varied in their susceptibility to infection outside of the biofilm condition, we measured the efficiency of plating for λ*cI*_857_, λΔ*cI,* and λvir on Δ*csgBA* (curli^−^), AR3110 (curli^+^), and csgD* (curli^++^) host strain backgrounds. This was performed by inoculating monoculture lawns of each strain in soft agar, followed by inoculation of a stock phage dilution series incubating overnight for plaque formation.

### Coinvasion of phages and lysogens into established biofilms

Sensitive biofilms of different strain backgrounds (Δ*csgBA* [curli^−^], AR3110 [curli^+^], and csgD* [curli^++^]) were grown for 72 h prior to addition of equal titers of phages (either λΔ*cI* or λvir) and lysogens (AR3110 curli^+^) (10^4^ PFU/µL and 10^4^ CFU/µL, respectively) for 2 h. We then reconnected the microfluidic flow cells to sterile M9 media and incubated for 120 h, imaging every 24 h to measure community composition over time. To measure whether the titers of invading phages and lysogens were changing over the course of the coinvasion, λ*cI*_857_ lysogens were incubated with either λvir or λΔ*cI* and the CFU and PFU titers were sampled every 15 min for 2 h.

### Microscopy and image analysis

All imaging was done using a Zeiss 980 line-scanning confocal microscope with a 40×/1.2 N.A. water objective. The mTurquoise2 protein fused to the capsids of λ*cI*_WT,_ λ*cI*_857_, λvir, and λΔ*cI* phages was excited with a 458 laser line (in separate experiments). The sfGFP protein expressed by the malK promoter was excited with a 488 laser line. The mKO2 protein that lysogenized *E. coli* expresses was excited with a 543 laser line. The mKate2 that *E. coli* expresses was excited with a 594 laser line. One image was taken to capture the dynamics for each biological replicate per chamber. Prior to export, images were processed by constrained iterative deconvolution in ZEN blue. Images were then imported into the BiofilmQ framework. Constitutive reporter signals were binarized using Otsu thresholding with a manual sensitivity parameter. To remove phages in free virion particle form from virocell biovolume calculations, voxel clusters that were smaller than 0.4 µm^3^ were removed from the analysis. For all analyses, a 3-dimensional grid was used to divide the segmented biovolumes into pseudo-cell cubes that measured 0.72 µm per side. Cell packing measurements combined the biovolume of all bacteria in a sample and calculated the fraction of total biovolume located within 6 μm of each segmented grid cube.

### Replication and statistical analysis

Biological replicates are indicated within figure legends. Biological replicates were drawn from distinct microfluidic chambers inoculated independently. Statistical significance was evaluated using Mann–Whitney *U* tests with Bonferroni corrections for multiple comparisons or one-sample Wilcoxon test against a null comparison value, indicated in figure legends. We chose nonparametric tests as the assumptions underlying parametric tests could not be assessed for our data. Data points within each figure represent independent biological replicates, and trend lines indicate median values. For time course plots, median value with interquartile ranges were plotted.

## Supporting information

S1 Fig **(A)** Population burst size of λ*cI*_857_ phages with mTurquoise2 capsid label, which is the fluorescent label used for all experiments (*n* = 4). **(B)** Spontaneous induction within biofilm monocultures of AR3110 curli^+^ *E. coli* lysogenized with λ*cI*_857_ or λ*cI*_WT_ (Mann–Whitney *U*-test, *n* = 4, 8). The data underlying this Figure can be found in S1 Data.
(PDF)

S2 Fig Virocell biovolume and frequency immediately after lytic induction within lysogenized *E. coli* AR3110 (curli^+^) or Δ*csgBA* (curli^−^). **(A)** Absolute volume of virocells (i.e., cells undergoing active lytic infection) following heat treatment of λ*cI*_857_ lysogens (Mann–Whitney *U*-test, *n* = 14–16). **(B)** Frequency of lytically inducing cells following heat treatment (Mann–Whitney *U*-test, *n* = 14–16). **(C)** Representative image of an AR3110 (curli^+^) biofilm following lytic induction. The data underlying this Figure can be found in S1 Data.
(PDF)

S3 Fig Quantification of uninfected biovolume, lysogenized biovolume, and virocell biovolume (i.e., cells undergoing active lytic infection) in *E. coli* biofilms inoculated with non-lysogenized *E. coli* and lysogenized *E. coli* at an initial ratio of 10:1. Here, lysogens carried prophages of λ*cI*_WT_, which induces the lytic cycle when its cI repressor is cleaved by host RecA. This is by contrast with experiments in Fig 1 of the main text, which were performed with lysogens carrying prophages of λ*cI*_857_, which induces lytic infection upon a temperature shift to 42°C. These experiments were performed as controls to document whether the dynamical patterns of phage infection and host lysogenization that were observed in the original experiments with λ*cI*_857_ would be recapitulated with λ*cI*_WT_. Here, instead of using heat to induce lytic propagation of prophages inoculated with uninfected hosts, biofilms were grown without disturbance, and λ*cI*_WT_ prophages induced lytic infection spontaneously without experimental manipulation. As for Fig 1 of the main text, the experiment was performed in **(A)** an AR3110 (curli^+^) *E. coli* strain background, and in **(B)** a Δ*csgBA* (curli^−^) strain background. The same qualitative population dynamics were observed here as for Fig 1B and 1C, albeit with delayed lytic propagation and lysogenization of new hosts in the Δ*csgBA* (curli^−^) treatment. We speculate that this is due to a lower overall degree of spontaneous lytic induction among λ*cI*_WT_ lysogens in comparison with the lytic induction of λ*cI*_857_ via the heat treatment manipulation for the experiments depicted in Fig 1 of the main text. The data underlying this Figure can be found in S1 Data.
(PDF)

S4 Fig Transcription of *malK* was active regardless of cell position or neighborhood cell packing within biofilms if maltose was the sole carbon source provided. **(A)** *malK* transcriptional reporter intensity (AU) within AR3110 curli^+^ biofilms grown in glucose or maltose, plotted as a function of cell packing (*n* = 4). **(B)** Representative image of *E. coli* grown in maltose, where the GFP-transcriptional *malK* reporter is active throughout the biofilm. **(C)** Representative image of *E. coli* grown in glucose, where the GFP-transcriptional *malK* reporter is inactive. **(D)** *malK* transcription (AU) as a function of cell packing in biofilms of the Δ*csgBA* curli^−^ strain in glucose or maltose (*n* = 4), or **(E)** in biofilms of the csgD* (curli^++^) strain (*n* = 5) in glucose or maltose. The data underlying this Figure can be found in S1 Data.
(PDF)

S5 Fig Comparison of efficacy of phage infection in the three strains used across all experiments in the study—Δ*csgBA* (curli^−^); AR3110 (curli^+^), and csgD* (curli^++^)—for each of the main phage variants used in the study—λ*cI*_857_, λΔ*cI*, and λvir. For each phage strain, no significant differences were observed in efficiency of plating across the three host bacterial different strains used for experiements in this study (*n* = 3, 4, Mann–Whitney *U* tests with bonferroni correction). The data underlying this Figure can be found in S1 Data.
(PDF)

S6 Fig Illustrative diagram for the design of experiments in Fig 2 of the main text. In separate experiments, *E. coli* with varying degrees of curli extracellular matrix production were inoculated and grown for 72 h in biofilm monoculture before being invaded with a mixture of lysogens and phage virions. The *E. coli* strain backgrounds included the double deletion mutant Δ*csgBA* (phenotype denoted curli^−^, shown in red), which cannot produce curli matrix protein; the parental *E. coli* strain AR3110 (phenotype denoted curli^+^, shown in purple), which produces curli matrix, and finally the *csg* promoter mutant csgD* (phenotype denoted curli^++^, shown in pink), which produces curli earlier and at higher rates than the parental AR3110 strain. Once grown, these separate biofilm growth chambers were invaded with a mixture of lysogens containing a λ*cI*_857_ prophage (shown in yellow) and virulent λ phage virions (small blue viruses) from one of two different phage strains: λΔ*cI*, which cannot superinfect lysogens, and λvir, which can superinfect lysogenized *E. coli*. After coinvasion, biofilms were tracked daily for 120 h for changes in uninfected and infected biovolume.
(PDF)

S7 Fig Population dynamics of λ*cI*_857_ lysogens and either (A) virulent phages that cannot superinfect lysogens (λΔ*cI*) or (B) virulent phages that can superinfect lysogens (λvir) in static culture over 2 h (*n* = 3). These experiments served as a control to determine whether the ratio of phages and lysogens could have changed within the syringes during the co-culture invasion experiments, whose results are shown in Fig 2 of the main text. On this 2 h time scale, no subtantial differences were observed between the compositions of the mixtures of lysogens with virulent non-superinfecting λΔ*cI* phages versus virulent and superinfecting λvir phages. The data underlying this Figure can be found in S1 Data.
(PDF)

S8 Fig Schematic of the microfluidic devices. This diagram illustrates 4 parallel chambers that are connected to inflow and outflow tubes through which media is driven at a constant rate. The vacuum port leads to a peripheral channel and is connected to a laboratory vacuum line to minimize introduction of air bubbles into the biofilm chambers.
(PDF)

S1 Data Underlying data. (XLSX)

## Abbreviation:

PDMS: poly-dimethylsiloxane
