# Supplementary material for: Biofilm spatial structure and superinfection immunity modulate inter-phage competition
Source: PLoS Biol. 2026 Mar 31;24(3):e3003737. doi: 10.1371/journal.pbio.3003737 (PMC13082703; doi:10.1371/journal.pbio.3003737)
Supplement: S1 Fig — (A) Population burst size of λcI857 phages with mTurquoise2 capsid label, which is the fluorescent label used for all experiments (n = 4). (B) Spontaneous induction within biofilm monocultures of AR3110 curli+ E. coli lysogenized with λcI857 or λcIWT (Mann–Whitney U-test, n = 4, 8). The data underlying this Figure can be found in S1 Data. (PDF) [file pbio.3003737.s001.pdf]

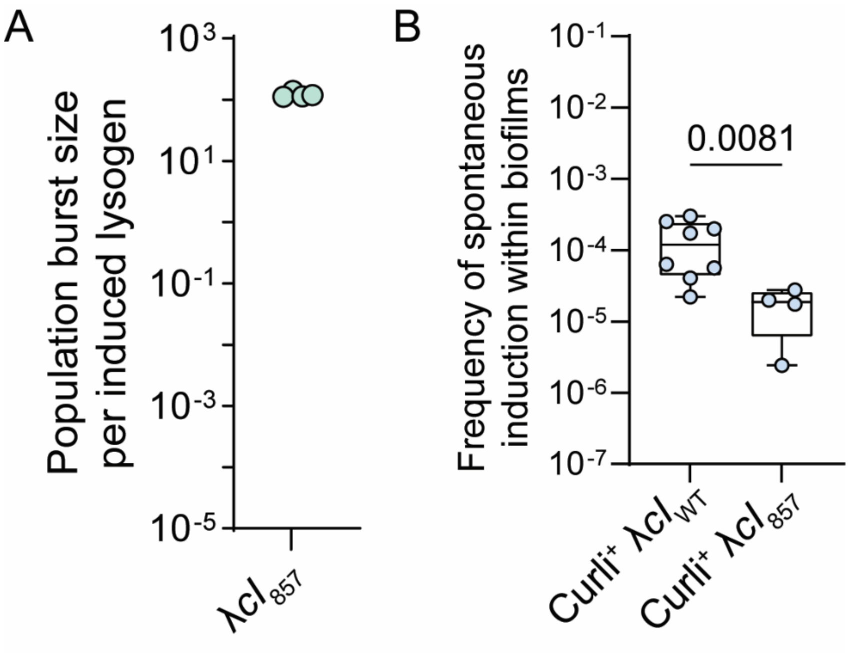

S1 Fig. – (A) Population burst size of λcI_{857} phages with mTurquoise2 capsid label, which is the fluorescent label used for visualizing phage virions in all experiments (n=4). (B) Spontaneous induction frequency within biofilm monocultures of AR3110 (curli$^+$) *E. coli* lysogenized with λcI_{857} or λcI_{WT} (Mann-Whitney U-test, n=4, 8).
