# Supplementary material for: Biofilm spatial structure and superinfection immunity modulate inter-phage competition
Source: PLoS Biol. 2026 Mar 31;24(3):e3003737. doi: 10.1371/journal.pbio.3003737 (PMC13082703; doi:10.1371/journal.pbio.3003737)
Supplement: S2 Fig — (A) Absolute volume of virocells (i.e., cells undergoing active lytic infection) following heat treatment of λcI857 lysogens (Mann–Whitney U-test, n = 14–16). (B) Frequency of lytically inducing cells following heat treatment (Mann–Whitney U-test, n = 14–16). (C) Representative image of an AR3110 (curli+) biofilm following lytic induction. The data underlying this Figure can be found in S1 Data. (PDF) [file pbio.3003737.s002.pdf]

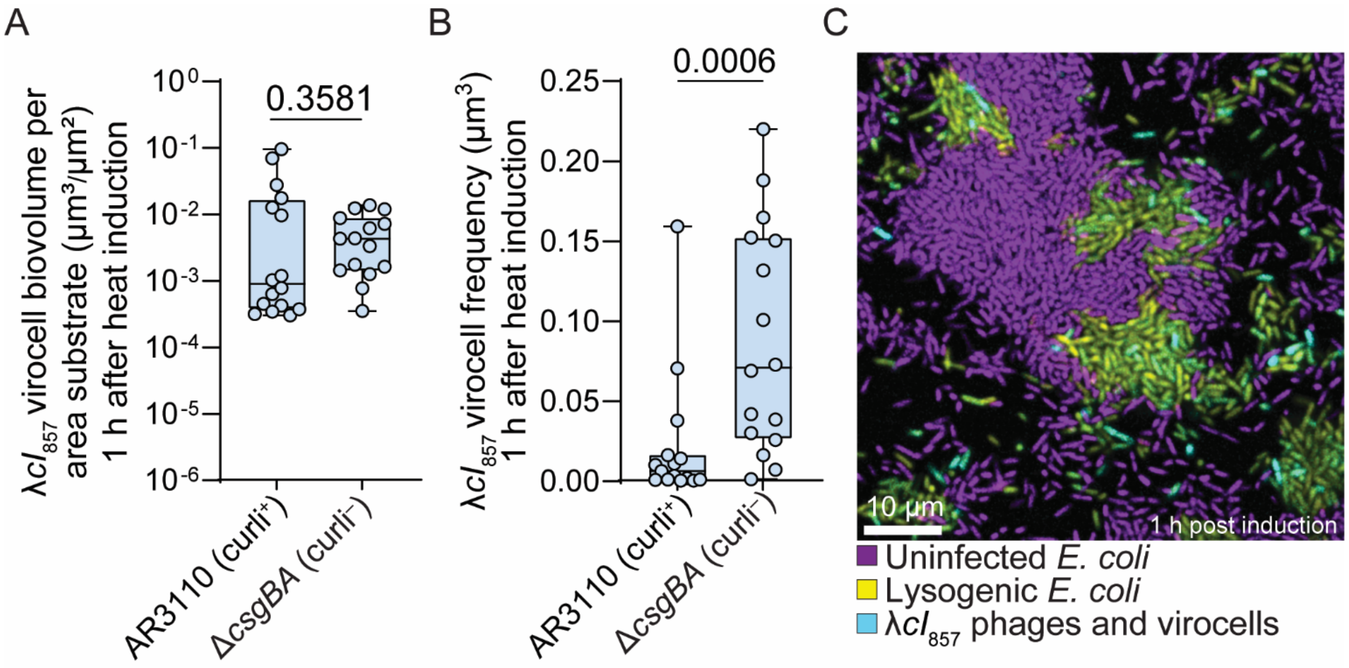

S2 Fig. – Virocell absolute biovolume and frequency immediately after lytic induction within lysogenized *E. coli* AR3110 (curli^{+}) or Δ*csgBA* (curli^{-}). (A) Absolute volume of virocells (i.e., cells undergoing active lytic infection) following heat treatment of λcI_{857} lysogens (Mann-Whitney U-test, n=14-16). (B) Frequency of lytically inducing cells following heat treatment (Mann-Whitney U-test, n=14-16). (C) Representative image of an AR3110 (curli^{+}) biofilm following lytic induction.
