# Supplementary material for: Biofilm spatial structure and superinfection immunity modulate inter-phage competition
Source: PLoS Biol. 2026 Mar 31;24(3):e3003737. doi: 10.1371/journal.pbio.3003737 (PMC13082703; doi:10.1371/journal.pbio.3003737)
Supplement: S3 Fig — Here, lysogens carried prophages of λcIWT, which induces the lytic cycle when its cI repressor is cleaved by host RecA. This is by contrast with experiments in Fig 1 of the main text, which were performed with lysogens carrying prophages of λcI857, which induces lytic infection upon a temperature shift to 42°C. These experiments were performed as controls to document whether the dynamical patterns of phage infection and host lysogenization that were observed in the original experiments with λcI857 would be recapitulated with λcIWT. Here, instead of using heat to induce lytic propagation of prophages inoculated with uninfected hosts, biofilms were grown without disturbance, and λcIWT prophages induced lytic infection spontaneously without experimental manipulation. As for Fig 1 of the main text, the experiment was performed in (A) an AR3110 (curli+) E. coli strain background, and in (B) a ΔcsgBA (curli−) strain background. The same qualitative population dynamics were observed here as for Fig 1B and 1C, albeit with delayed lytic propagation and lysogenization of new hosts in the ΔcsgBA (curli−) treatment. We speculate that this is due to a lower overall degree of spontaneous lytic induction among λcIWT lysogens in comparison with the lytic induction of λcI857 via the heat treatment manipulation for the experiments depicted in Fig 1 of the main text. The data underlying this Figure can be found in S1 Data. (PDF) [file pbio.3003737.s003.pdf]

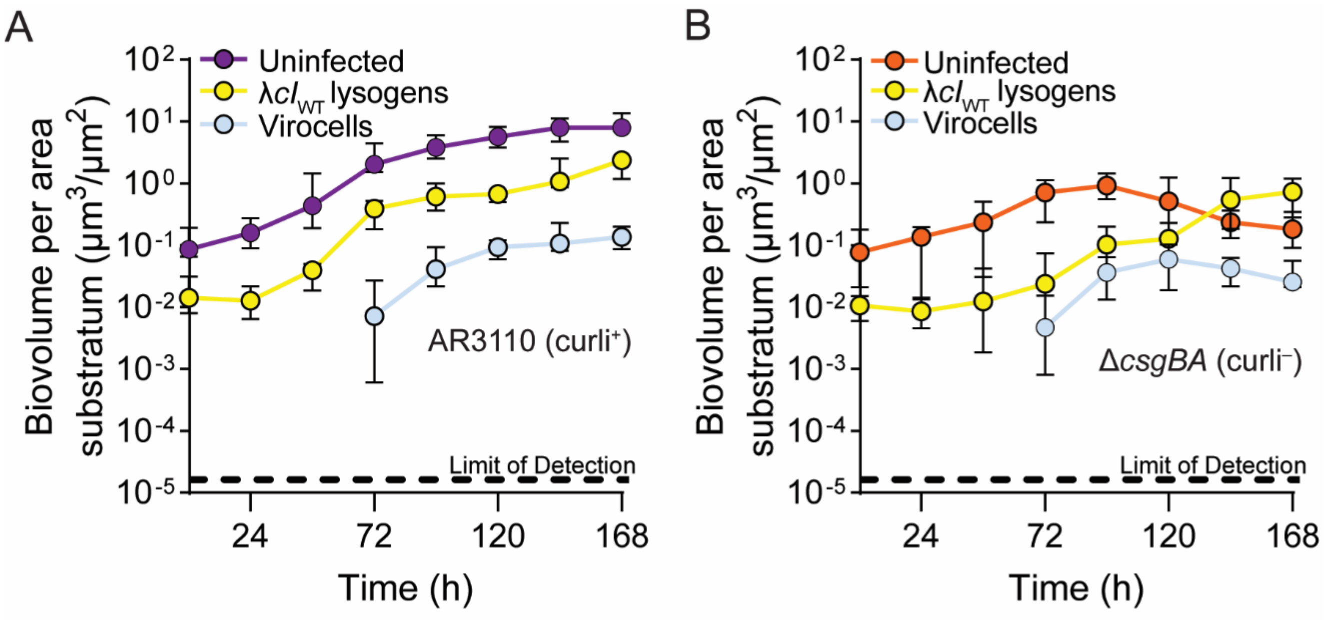

S3 Fig. – Quantification of uninfected biovolume, lysogenized biovolume, and virocell biovolume (i.e., cells undergoing active lytic infection) in *E. coli* biofilms inoculated with non-lysogenized *E. coli* and lysogenized *E. coli* at an initial ratio of 10:1. Here, lysogens carried prophages of λcI_{WT}, which induces the lytic cycle when its cI repressor is cleaved by host RecA. This is by contrast with experiments in Figure 1 of the main text, which were performed with lysogens carrying prophages of λcI_{857}, which induces lytic infection upon a temperature shift to 42C°. These experiments were performed as controls to document whether the dynamical patterns of phage infection and host lysogenization that were observed in the original experiments with λcI_{857} would be recapitulated with λcI_{WT}. Here, instead of using heat to induce lytic propagation of prophages inoculated with uninfected hosts, biofilms were grown without disturbance, and λcI_{WT} prophages induced lytic infection spontaneously without experimental manipulation. As for Figure 1 of the main text, the experiment was performed in (A) an AR3110 ($curli^+$) *E. coli* strain background, and in (B) a Δ*csgBA* ($curli^–$) strain background. The same qualitative population dynamics were observed here as for Figure 1 B and C, albeit with delayed lytic propagation and lysogenization of new hosts in the Δ*csgBA* ($curli^–$) treatment. We speculate that this is due to a lower overall degree of spontaneous lytic induction among λcI_{WT} lysogens in comparison with the lytic induction of λcI_{857} via the heat treatment manipulation for the experiments depicted in Figure 1 of the main text.
