# Supplementary material for: Biofilm spatial structure and superinfection immunity modulate inter-phage competition
Source: PLoS Biol. 2026 Mar 31;24(3):e3003737. doi: 10.1371/journal.pbio.3003737 (PMC13082703; doi:10.1371/journal.pbio.3003737)
Supplement: S4 Fig — (A) malK transcriptional reporter intensity (AU) within AR3110 curli+ biofilms grown in glucose or maltose, plotted as a function of cell packing (n = 4). (B) Representative image of E. coli grown in maltose, where the GFP-transcriptional malK reporter is active throughout the biofilm. (C) Representative image of E. coli grown in glucose, where the GFP-transcriptional malK reporter is inactive. (D) malK transcription (AU) as a function of cell packing in biofilms of the ΔcsgBA curli− strain in glucose or maltose (n = 4), or (E) in biofilms of the csgD* (curli++) strain (n = 5) in glucose or maltose. The data underlying this Figure can be found in S1 Data. (PDF) [file pbio.3003737.s004.pdf]

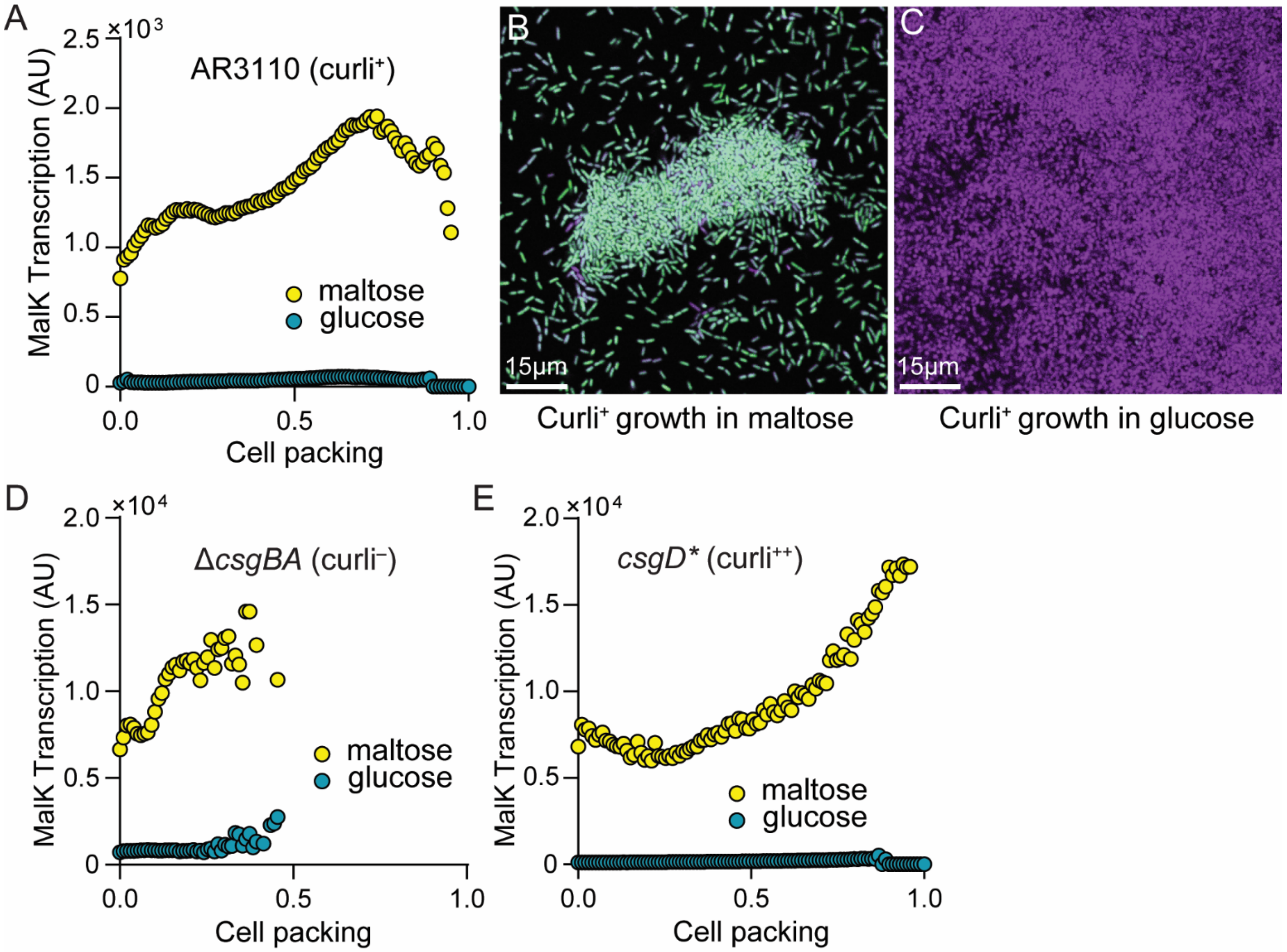

S4 Fig. – Transcription of *malK* was active regardless of cell position or neighborhood cell packing within biofilms if maltose was the sole carbon source provided. (A) *malK* transcriptional reporter intensity (AU) within AR3110 curli$^+$ biofilms grown in glucose or maltose, plotted as a function of cell packing (n=4). (B) Representative image of *E. coli* grown in maltose, where the GFP-transcriptional *malK* reporter is active throughout the biofilm. (C) Representative image of *E. coli* grown in glucose, where the GFP-transcriptional *malK* reporter is inactive. (D) *malK* transcription (AU) as a function of cell packing in biofilms of the Δ*csgBA* curli$^-$ strain in glucose or maltose (n=4), or (E) in biofilms of the *csgD** (curli^{++}) strain (n=5) in glucose or maltose.
