# Supplementary material for: Biofilm spatial structure and superinfection immunity modulate inter-phage competition
Source: PLoS Biol. 2026 Mar 31;24(3):e3003737. doi: 10.1371/journal.pbio.3003737 (PMC13082703; doi:10.1371/journal.pbio.3003737)
Supplement: S5 Fig — For each phage strain, no significant differences were observed in efficiency of plating across the three host bacterial different strains used for experiements in this study (n = 3, 4, Mann–Whitney U tests with bonferroni correction). The data underlying this Figure can be found in S1 Data. (PDF) [file pbio.3003737.s005.pdf]

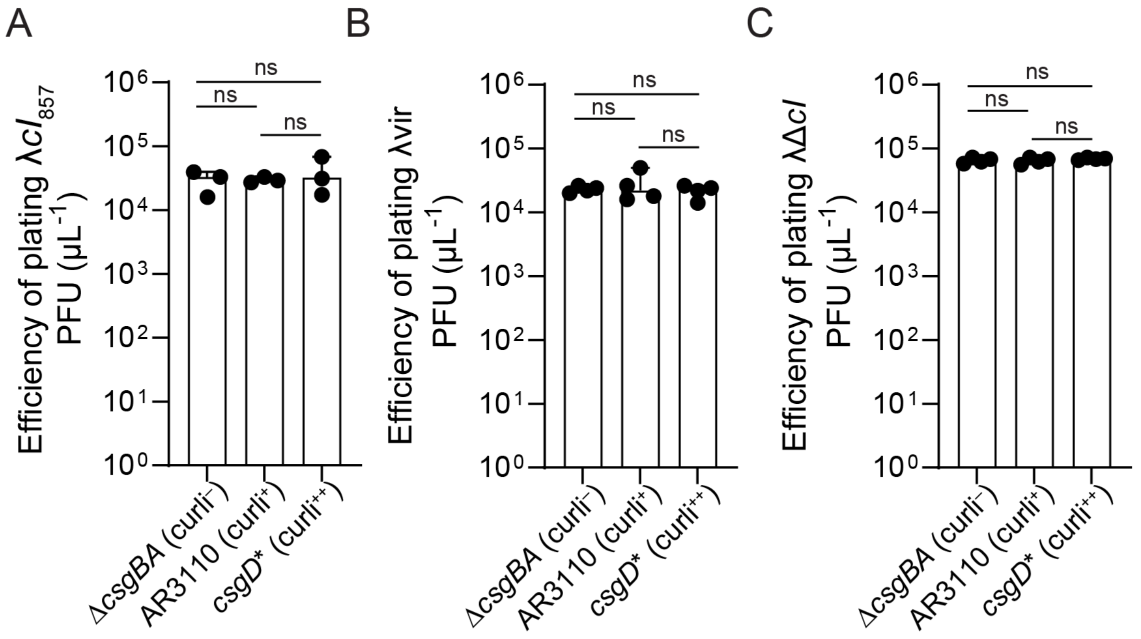

S5 Fig. – Comparison of efficacy of phage infection in the three strains used across all experiments in the study – Δ*csgBA* (curli$^-$); AR3110 (curli$^+$), and *csgD** (curli^{++}) – for each of the main phage variants used in the study – λcI_{857}, λΔ*cI*, and λvir. For each phage strain, no significant differences were observed in efficiency of plating across the three host bacterial different strains used for experiements in this study (n=3, 4, Mann-Whitney U tests with bonferroni correction).
