# Supplementary material for: Biofilm spatial structure and superinfection immunity modulate inter-phage competition
Source: PLoS Biol. 2026 Mar 31;24(3):e3003737. doi: 10.1371/journal.pbio.3003737 (PMC13082703; doi:10.1371/journal.pbio.3003737)
Supplement: S6 Fig — In separate experiments, E. coli with varying degrees of curli extracellular matrix production were inoculated and grown for 72 h in biofilm monoculture before being invaded with a mixture of lysogens and phage virions. The E. coli strain backgrounds included the double deletion mutant ΔcsgBA (phenotype denoted curli−, shown in red), which cannot produce curli matrix protein; the parental E. coli strain AR3110 (phenotype denoted curli+, shown in purple), which produces curli matrix, and finally the csg promoter mutant csgD* (phenotype denoted curli++, shown in pink), which produces curli earlier and at higher rates than the parental AR3110 strain. Once grown, these separate biofilm growth chambers were invaded with a mixture of lysogens containing a λcI857 prophage (shown in yellow) and virulent λ phage virions (small blue viruses) from one of two different phage strains: λΔcI, which cannot superinfect lysogens, and λvir, which can superinfect lysogenized E. coli. After coinvasion, biofilms were tracked daily for 120 h for changes in uninfected and infected biovolume. (PDF) [file pbio.3003737.s006.pdf]

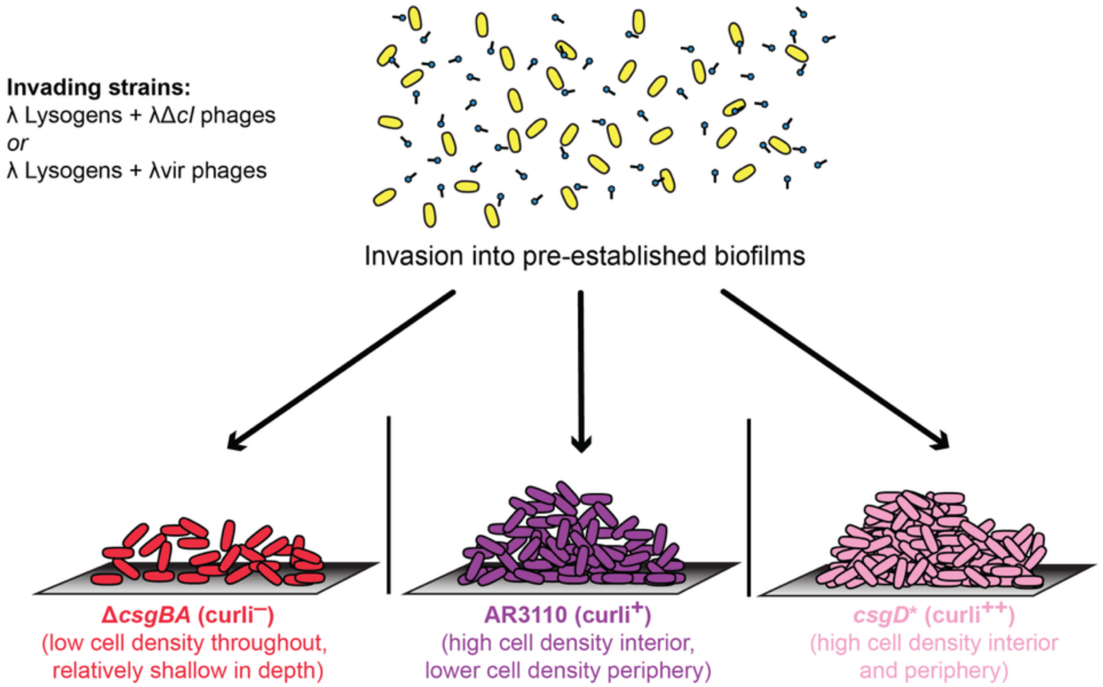

S6 Fig. – Illustrative diagram for the design of experiments in Figure 2 of the main text. In separate experiments, *E. coli* with varying degrees of curli extracellular matrix production were inoculated and grown for 72 h in biofilm monoculture before being invaded with a mixture of lysogens and phage virions. The *E. coli* strain backgrounds included the double deletion mutant Δ*csgBA* (phenotype denoted curli^{-}, shown in red), which cannot produce curli matrix protein; the parental *E. coli* strain AR3110 (phenotype denoted curli^{+}, shown in purple), which produces curli matrix, and finally the *csg* promoter mutant *csgD** (phenotype denoted curli^{++}, shown in pink), which produces curli earlier and at higher rates than the parental AR3110 strain. Once grown, these separate biofilm growth chambers were invaded with a 1:1 mixture of lysogens containing a λcI_{857} prophage (shown in yellow) and virulent λ phage virions (small blue viruses) from one of two different phage strains: λΔ*cI*, which cannot superinfect lysogens, and λvir, which can superinfect lysogenized *E. coli*. After coinvasion, biofilms were tracked daily for 120 h for changes in uninfected and infected biovolume.
