# Supplementary material for: Biofilm spatial structure and superinfection immunity modulate inter-phage competition
Source: PLoS Biol. 2026 Mar 31;24(3):e3003737. doi: 10.1371/journal.pbio.3003737 (PMC13082703; doi:10.1371/journal.pbio.3003737)
Supplement: S7 Fig — These experiments served as a control to determine whether the ratio of phages and lysogens could have changed within the syringes during the co-culture invasion experiments, whose results are shown in Fig 2 of the main text. On this 2 h time scale, no subtantial differences were observed between the compositions of the mixtures of lysogens with virulent non-superinfecting λΔcI phages versus virulent and superinfecting λvir phages. The data underlying this Figure can be found in S1 Data. (PDF) [file pbio.3003737.s007.pdf]

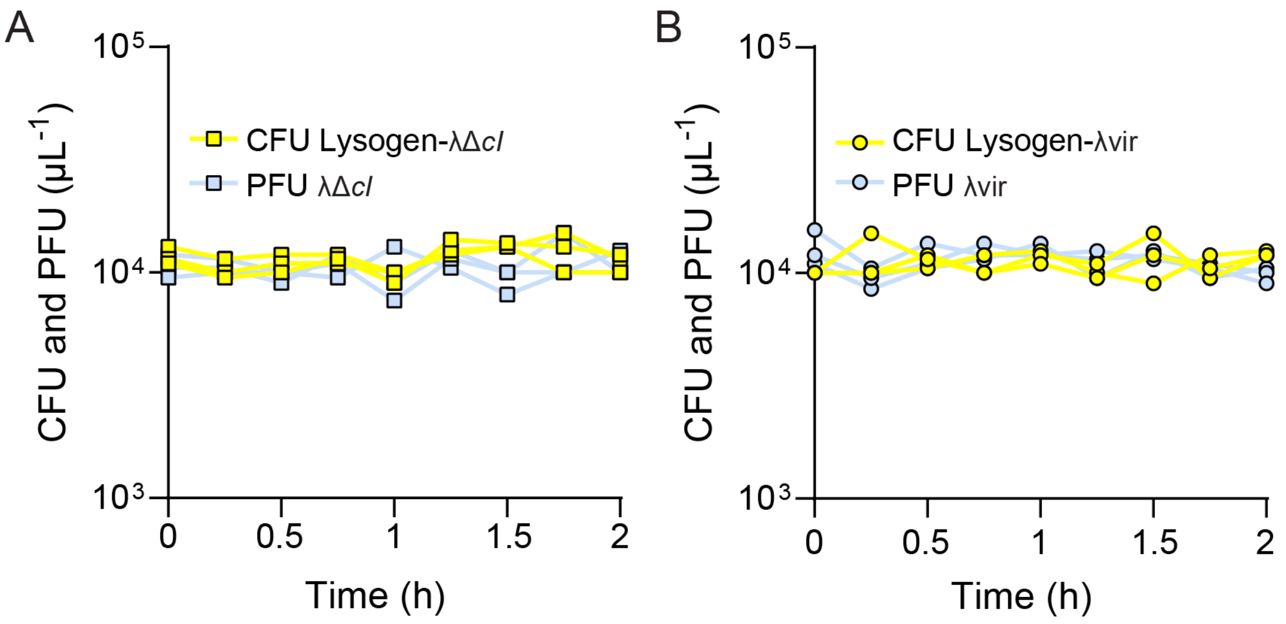

S7 Fig. – Population dynamics of λ*cI*$_{857}$ lysogens and either (A) virulent phages that cannot superinfect lysogens (λΔ*cI*) or (B) virulent phages that can superinfect lysogens (λvir) in static culture over 2 h (n=3). These experiments served as a control to determine whether the ratio of phages and lysogens could have changed within the syringes during the co-culture invasion experiments, whose results are shown in Figure 2 of the main text. On this 2 h time scale, no subtantial differences were observed between the compositions of the mixtures of lysogens with virulent non-superinfecting λΔ*cI* phages versus virulent and superinfecting λvir phages.
