# Supplementary material for: Biofilm spatial structure and superinfection immunity modulate inter-phage competition
Source: PLoS Biol. 2026 Mar 31;24(3):e3003737. doi: 10.1371/journal.pbio.3003737 (PMC13082703; doi:10.1371/journal.pbio.3003737)
Supplement: S8 Fig — This diagram illustrates 4 parallel chambers that are connected to inflow and outflow tubes through which media is driven at a constant rate. The vacuum port leads to a peripheral channel and is connected to a laboratory vacuum line to minimize introduction of air bubbles into the biofilm chambers. (PDF) [file pbio.3003737.s008.pdf]

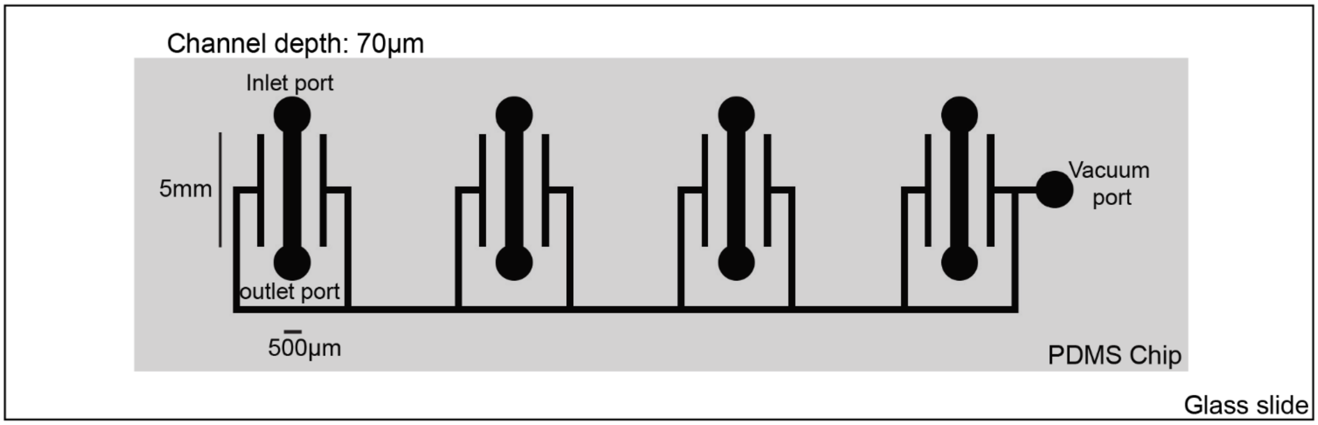

S8 Fig. – Schematic of the microfluidic devices. This diagram illustrates 4 parallel chambers that are connected to inflow and outflow tubes through which media is driven at a constant rate. The vacuum port leads to a peripheral channel and is connected to a laboratory vacuum line to minimize introduction of air bubbles into the biofilm chambers.
